# Review of Influenza Virus Vaccines: The Qualitative Nature of Immune Responses to Infection and Vaccination Is a Critical Consideration

**DOI:** 10.3390/vaccines9090979

**Published:** 2021-09-01

**Authors:** Lily Chan, Kasra Alizadeh, Kimia Alizadeh, Fatemeh Fazel, Julia E. Kakish, Negar Karimi, Jason P. Knapp, Yeganeh Mehrani, Jessica A. Minott, Solmaz Morovati, Amira Rghei, Ashley A. Stegelmeier, Sierra Vanderkamp, Khalil Karimi, Byram W. Bridle

**Affiliations:** 1 Department of Pathobiology, Ontario Veterinary College, University of Guelph, Guelph, ON N1G 2W1, Canada; lchan12@uoguelph.ca (L.C.); ffazel@uoguelph.ca (F.F.); jkakish@uoguelph.ca (J.E.K.); jknapp03@uoguelph.ca (J.P.K.); ymehrani@uoguelph.ca (Y.M.); minott@uoguelph.ca (J.A.M.); arghei@uoguelph.ca (A.R.); astegelm@uoguelph.ca (A.A.S.); vanderka@uoguelph.ca (S.V.); 2 Department of Pharmaceutical Sciences, College of Pharmacy, University of Illinois at Chicago, Chicago, IL 60612, USA; kaliza2@uic.edu; 3 Department of Diagnostic Medicine & Pathobiology, College of Veterinary Medicine, Kansas State University, Manhattan, KS 66506, USA; kimia@vet.k-state.edu; 4 Department of Clinical Science, School of Veterinary Medicine, Ferdowsi University of Mashhad, Azadi Square, Mashhad 91779-48974, Iran; n.karimi@mail.um.ac.ir; 5 Division of Biotechnology, Department of Pathobiology, School of Veterinary Medicine, Shiraz University, Shiraz 71441-69155, Iran; s.morovati@shirazu.ac.ir

**Keywords:** influenza virus, trained immunity, mRNA vaccine, sex-mediated antiviral response

## Abstract

Influenza viruses have affected the world for over a century, causing multiple pandemics. Throughout the years, many prophylactic vaccines have been developed for influenza; however, these viruses are still a global issue and take many lives. In this paper, we review influenza viruses, associated immunological mechanisms, current influenza vaccine platforms, and influenza infection, in the context of immunocompromised populations. This review focuses on the qualitative nature of immune responses against influenza viruses, with an emphasis on trained immunity and an assessment of the characteristics of the host–pathogen that compromise the effectiveness of immunization. We also highlight innovative immunological concepts that are important considerations for the development of the next generation of vaccines against influenza viruses.

## 1. Introduction

This review provides an overview of influenza viruses and how they have remained a global public health concern since the 1918 influenza pandemic (the Spanish flu), which was caused by the H1N1 influenza A virus, and essentially introduced the influenza virus to the world [1]. Mechanisms in both the influenza virus and the host’s immune system can result in viral evasion and infection of the host. A vaccination is the most common method to prevent diseases associated with influenza viruses. However, current influenza vaccination strategies have substantial limitations. Vaccines represent an opportunity to optimize the way in which the host’s immune system will respond to pathogens, in both the magnitude and the qualitative nature of the response. There are important considerations in host immune responses to influenza infection and vaccination, which have been largely ignored in vaccine design and recent developments in the immunology field; they should be investigated further to optimize vaccine efficacy. This review will discuss qualitative aspects of host immunity against influenza viruses that have received less attention in comparison to quantitative features, and how they may play a role in the effectiveness of the protection against influenza conferred by the vaccination. We also briefly review the importance of messenger ribonucleic acid (mRNA)-based vaccines (as a recent vaccine platform), and how some studies focus on the design and development of mRNA vaccines against influenza.

## 2. Types of Influenza Viruses

Influenza viruses are members of the family Orthomyxoviridae, which comprises enveloped viruses with varied antigenic characteristics. Influenza viruses are spherical or filamentous; the genome contains segmented negative-sense single-stranded RNA. The segmented RNA genome of the influenza virus family accelerates antigenic variability [2]. There are four main genera in this family: types A, B, C, and D. Types A and B are clinically relevant to humans and are responsible for most of the flu outbreaks, while type C normally causes a milder upper respiratory infection in humans [3,4]. In addition to humans, the influenza A virus (IAV) is able to infect a number of animals, including pigs, dogs, horses, and birds [5]. Type D influenza virus has not been shown to cause illness in humans [1]. Therefore, this review will mainly focus on type A and B influenza viruses.

Hemagglutinin (HA or H) and neuraminidase (NA or N) are the two major glycoproteins on the surface of the influenza virus, playing key roles in pathogenesis of the infection [6]. Genetic and antigenic characteristics of HA and NA determine several subtypes for this virus. There are 18 HA (H1–H18) and eleven NA (N1–N11) subtypes described thus far [1]. The nomenclature follows a H(x)N(y) pattern in which the host, geographical location of the first isolation, strain number, and year of isolation are mentioned when they are identified [7,8].

The influenza B virus has a similar viral structure to type A, but it is not further divided into subtypes. Some minor antigenic variations have been reported since 1970 in this virus, which has led to two antigenically detectable lineages, Victoria and Yamagata [9].

Antigenic shifts and antigenic drifts take place when there are major or minor changes in the characteristics of surface glycoproteins in type A and B influenza viruses. Antigenic shifts are associated with the epidemics and pandemics of IAV, whereas antigenic drifts are responsible for more localized outbreaks [10]. Antigenic shifts occur when there is an exchange in genomic segments at the time of a simultaneous infection of a cell by two different influenza A viruses. This in turn might bring about a selective advantage of the new virus compared to the parent viruses. An example is the occurrence of the influenza A/H2N2 subtype in 1957, which overthrew the subtype that was dominant at the time (influenza A/H1N1 virus) [10]. In the case of type B influenza viruses, variations also occur through mechanisms, such as insertion and deletion [11,12]. Influenza C virus is the third human influenza virus type and consists of seven genome segments. The surface of the influenza C virus is characterized by a single spike protein named hemagglutinin-esterase-fusion glycoprotein [13], which conveys both receptor-binding and -destroying functions [14]. Hemagglutinin-esterase-fusion glycoprotein is also present in influenza D viruses, where it plays a major role in binding to trachea sections from human, swine, and bovine origin [15].

## 3. Antigenic Drift in Influenza Virus

Influenza infections arise in the human population through seasonal epidemics, occurring during various cyclical periods in different regions across the globe. Currently, approved influenza vaccines rely on the immune system to target immunity against the viral HA protein. Seasonal vaccines are critical in protecting the population against influenza and in-depth analyses and redesign must be performed regularly to keep up with the constantly evolving circulating influenza stains. The fast-paced changes in circulating strains are due to antigenic drift.

Antigenic drift is the result of mutations occurring in the surface proteins and other viral proteins due to error-prone viral RNA-dependent RNA polymerase lacking the ability to proofread during viral replication [16,17]. These amino acid changes in both the HA and the NA surface glycoproteins are key factors in antigenic drift, as these substitutions lead to immune evasion of the virus and allow the virus to propagate in a population and emerge as a novel epidemic strain [18]. These continuous changes in the virus lead to the virus existing as quasispecies. Mapping and sequencing analysis of the surface glycoproteins of escape mutants has helped define five antigenic sites on the HA glycoprotein, named A–E [19]. Of the surface proteins, the HA glycoprotein is considered the primary focus of antigenic drift, which may be an area of oversight in vaccine evaluation and formulation as it is the primary focus in vaccine design.

With each viral particle entering a host cell for replication, these changes at the amino acid level result in the gradual accumulation of mutations, brought upon by selective pressure of the host immune system or antiviral drugs. Since the discovery of the A/Hong Kong/1/1968 (H3N2) influenza A virus, there has been a redesign of the seasonal vaccines at least once a year, from November 1998 to April 1999 [20], to the 2021–2022 [21] quadrivalent vaccine. Furthermore, antigenic drift in influenza A/H3N2 has illustrated how some amino acid substitutions can result in little to no escape variants, while some substitutions can create the emergence of new subspecies that have the potential to cause an epidemic. The influenza A/H3N2 stain from 2005 to 2006 showed a single point-mutation at position 31 of the M2 protein, a transmembrane ion channel that equilibrates pH across the viral membrane upon entry into a host cell [22]. This mutation to the M2 protein resulted in a drastic increase in amantadine resistance, from 2 to 12% to greater than 91%, rendering the antiviral inadequate in inhibiting influenza replication [23]. These escape mutants will not be the dominant emerging pandemic strain during each season, but they have the possibility of gaining a single mutation that could escape the host’s immune system as well as antiviral treatments and vaccines. The association between antigenic drift and the influenza vaccine design occurs through natural evolution of the virus and the decisions by global public health officials.

## 4. Antigenic Shift in Influenza Virus

One major challenge for the development of influenza vaccines is the persistent alterations in the virus. Antigenic transformation occurs in influenza viruses through two processes, called antigenic drifts and antigenic shifts, which occur in the surface glycoproteins. Both mechanisms help the virus evade host immune responses, which results in complications for vaccination efficacy [24]. Contrary to antigenic drift, which is accompanied by minor gradual changes, an antigenic shift is the exchange of the entire genes that encode HA and/or NA [25]. The segmented nature of the influenza virus genome provides the possibility for gene segment exchange between viruses when a single cell is infected simultaneously with diverse viral particles [24,26]. In other words, an antigenic shift is the reassortment and presentation of new HA, NA, or both gene segments from different strains of influenza viruses that circulate among humans and animals [27].

Antigenic shift occurs exclusively in IAVs due to their genomic variations across multiple animal species, which serve as the sources of viruses with different antigenicity [28]. On the other hand, influenza B viruses do not undergo antigenic shifts because non-human animal hosts have not been identified for these viruses [24]. Overall, the process of antigenic shift leads to the emergence of novel IAVs in human populations that would have limited immunity and can cause problems in vaccination efficacy as well as elevated transmission, which has the potential to lead to a flu pandemic, as has been the result in the past.

## 5. Cross-Species Transmission of Influenza Virus

The emergence of pandemic influenza virus strains and new seasonal viruses have drawn the attention of scientists to the issue of cross-species transmission. There is a need to advance the understanding of how bird and animal influenza viruses can cross the species barrier and expand their host-range. Prevention of outbreaks of human influenza is dependent on strategies for controlling the disease in birds and animals, which can spread the virus to humans (especially considering the difficulty in obtaining complete protection against the numerous IAV variants). Therefore, slowing or blocking the virus spillover across species through vaccination is more achievable than designing a vaccine against all rapidly mutating viruses. As an example of controlling the disease in birds, Hajam et al. recently reported on the generation of protective immunity against avian influenza viruses in chickens with an mRNA vaccine packaged in chitosan nanoparticles [29].

The emergence of new zoonotic diseases arose with the beginning of the agriculture revolution and animal domestication [30]. It was accompanied by the significant growth in population size and an increasing the contact rate between humans and livestock. These events were the keystone of infectious disease spillover across species, including IAV infection of humans and a broad range of animals. Wild aquatic birds are considered the major reservoirs of IAV [31]. They do not appear to show the clinical signs of the disease, but they transmit the infection to more susceptible birds or mammals.

The transmission of IAVs among species relies on several host, viral, and epidemiological factors affecting virus–host cell interplay. For instance, due to the lack of exonuclease proofreading activity of RNA polymerase, RNA virus replication is error-prone [32], and this characteristic accelerates the mutation rate of RNA viruses including influenza and generates a heterogeneous population of mutated viruses [33]. Subsequently, genetic diversity increases the flexibility of the virus for adaptation to new hosts. The genetic changes are usually exerted by the selective pressure of the host immune system. However, the replication efficiency of the virus in a new host is dependent on the engagement of the virus by its specific cell surface receptors.

### 5.1. Intermediate Hosts

#### 5.1.1. Terrestrial Birds

Wild waterfowl bird viruses are considered the origin of all mammalian IAVs [31]. However, mutation of the virus in an intermediate host appears to be essential for the adaptation of avian strains to infect humans. Terrestrial birds, such as chickens, quail, and turkeys, can serve as intermediate hosts for avian-to-human adaptation [34,35].

#### 5.1.2. Pigs

Pigs have been long identified as the most likely intermediate hosts for adaptation of the avian influenza virus [36,37,38,39,40,41,42,43,44,45,46,47,48,49]. As per the hypothesis of ‘mixing vessel’, pigs can serve as a host for genetic reassortment among avian, swine, and human influenza strains. Accordingly, all of these viruses can replicate and complete their life cycles in pigs co-infected by two or more IAV strains and generate new variants of the virus. Several studies imply that the H3N2, H1N1, H3N1, and H1N2 subtypes are “reassortant” strains that emerged from pigs [40,50,51,52,53,54,55,56,57,58,59,60,61,62]. The 2009 H1N1 pandemic strain created through the mixing vessel mechanism is a complex virus containing RNA segments from influenza strains of avian, swine, and human origin [63,64]. Adaptation of IAV to humans can be mediated in pigs without any reassortment events [48,65,66]. Since pigs are recognized as the main source of the cross-species transmission of IAV, more research emphasis should be placed on developing effective vaccines for swine, to promote human health and safety.

#### 5.1.3. Horses

Horses were the source of continuous transmission of H3N8 and H7N7 influenza outbreaks in 1872 [65], 1956 [66], and 1963 [67], in which the viruses originated from an avian-like influenza strain, as well as other IAVs. In addition to isolation of H3N8 equine influenza virus from camels [68] and pigs [69], it was established for the first time in dogs in 1999 [70]. Since then, multiple H3N8 equine-to-canine host-jump incidences have been reported worldwide [71,72,73,74]. Indeed, some reassortment also occurred between equine H3N8 and H7N7 strains in the 1960s, which quickly disappeared during the 1970s [75].

#### 5.1.4. Dogs

Two well-known canine influenza viruses, H3N8 and H3N2, originated directly from equine and avian strains, respectively. Although no reassortment has been observed between H3N8 canine and other influenza viruses, it has been seen with H3N2 canine influenza viruses [76,77,78]. Furthermore, H3N8 canine influenza virus transmission to horses has not been verified. Based on some studies, cats [79] and ferrets [80,81] have been infected with canine strains. Nevertheless, given the self-limiting nature of the virus and the close relationship between dogs and people, the spread of the canine influenza virus to humans, particularly H3N2, is a matter of potential concern.

#### 5.1.5. Bats

Currently, bats are recognized as a source of new infectious viruses due to their potential role in the coronavirus disease 2019 pandemic. To date, studies show that bat IAVs are phylogenetically distinct from their mammal-derived counterparts. It had been concluded that they are unlikely to serve as a notable reservoir for influenza virus transmission to mammals [82,83]. However, more recent research has identified that bat influenza viruses could infect cells from a variety of species by using major histocompatibility complex II molecules as entry receptors [84]. Specifically, it was shown that cells from humans, pigs, chickens, and mice were susceptible to infection. This reopens the debate about whether bat influenza viruses could promote novel reassortants with the potential to infect humans. An influenza virus that causes disease in humans and that uses a molecule unique to antigen-presenting cells to gain entry could be particularly dangerous as it could potentially impair the induction of adaptive immune response.

### 5.2. Molecular Determinants of Species Specificity

Generally, mammalian-adapted influenza viruses are known as species-specific viruses. However, antigenic drift generates new epitopes or glycosylation sites, which can alter the viral tropism. For example, in the 1968 Hong-Kong pandemic, E → L and G → S amino acid substitutions at residues 226 and 228, respectively, of the HA protein switched the receptor-binding preference of the H3 subtype of the virus from avian receptors (α2,3-linked sialic acid) to human receptors (α2,6-linked sialic acid) [85,86,87]. Likewise, replacement of glutamic acid by aspartic acid (E → D substitution) at residue 190 was associated with an alteration of receptor-binding affinity of the H1 strain from avian to swine and human cells [86,87]. Antigenic shift by exchanging the large RNA segments among two or more subtypes of IAVs could also lead to the emergence of novel strains that gain the ability to replicate efficiently within new host species that were not previously susceptible [31,68,88,89,90,91,92,93,94,95,96,97,98]. It is an accepted notion that the last three pandemics of IAVs are reassorted descendants of the 1918 H1N1 strain [99].

The number and position of glycosylation sites in HA proteins are other factors determining the host range of the virus. The presence of one N-linked oligosaccharide at position 63 in human lineage viruses and the absence of one or two glycosylation sites in the mouse-adapted H3 strain are examples of different patterns of glycosylation altering the IAV receptor tropism [43]. Length and amino acid sequence of NA are correlated with pathogenicity and species specificity of the IAV, as well [71,100,101]. Viruses with a short NA stalk, such as the highly pathogenic avian influenza H5N1, show more virulence [102,103]. Interestingly, modifications of HA and NA often occur simultaneously, providing suitable conditions for the effective replication of the virus in the new host.

Polymerase complex [100,101] and internal proteins [70,98,104,105] can also determine the host tropism of the virus. The temperature of the replication site, mutation at key residues of the proteins, and their interaction with host cell components are factors that can affect the species specificity of IAV.

Some competitive inhibitors can restrict the cross-species transmission of IAV. α2-macroglobulin, a conserved effector molecule of the innate immune system of mammals, interferes with the infection of Madin–Darby canine kidney cells by the human H3 strain of influenza virus [106]. Similarly, SA residues on porcine surfactant protein D restricts the human-to-swine transmission of influenza viruses [107]. These components can be deployed for designing new vaccines to protect mammals and birds against the disease and concurrent spread of the virus between species.

## 6. Current Influenza Vaccines

The immune system encompasses an array of potent effector mechanisms. For many people, their natural immune responses are sufficient at clearing viral infections. The large spectrum of innate and adaptive effector cells and molecules can often prevent influenza or limit it to a mild, transient disease. This concept of naturally acquired immunity has been extensively reviewed elsewhere [104,105,108,109], although the constant emergence of novel variants renders natural immunity largely irrelevant after a relatively short period of time. Some individuals are inherently at risk of moderate to severe (and, especially, potentially fatal) influenza. This includes elderly individuals experiencing immunosenescence, very young individuals whose immune systems are still maturing, and those who are immunosuppressed. This is why vaccines represent an excellent strategy to confer some degree of protection.

The most important targets for influenza vaccines have been the viral membrane surface proteins, HA and NA. Hemagglutinin enables initial binding of the virus to the host cell by attachment to sialic acid, as well as fusion of the virus and host membranes for the release of the viral core into the host cell for viral replication. The HA protein is composed of a head and a stalk domain. The head domain is the primary target of antibodies (Abs) that confer immunity to influenza viruses by inhibiting their binding to host cells [110]. Neuraminidase removes sialic acid from viral proteins and is important in the detachment and spread of the virus. Antibodies to NA cause an aggregation of viral particles on the cell surface, reducing their ability to spread [110].

Like any other infection, innate immunity is critical in suppressing viral infections. However, inactivated vaccines are weak in their ability to elicit an innate immune response [111]. That being said, it has been shown that ultraviolet-inactivated avian influenza virus can trigger the activation of interferon (IFN)-inducible genes and cytokine production upon binding to human cells [112]. The primary immune response to immunization with the inactivated influenza virus is the production of Abs against surface proteins, such as the head domain of the HA protein and the NA protein. The former is thought to be the primary mediator of the immunity conferred by the current inactivated vaccines. As a result, the HA content of the inactivated vaccines is accurately measured and standardized [1]. Unlike HA, NA content of such vaccines is not quantified, and only a subset of NA Abs with a specific epitope have been studied [1]. The fact that NA can elicit protective Abs, some of which can even confer cross-reactive immunity [113], warrants the need for more research into the better use of this protein in vaccines.

Three types of influenza vaccines are currently licensed for use worldwide: inactivated vaccines, live attenuated vaccines, and recombinant HA vaccines. In each dose of the seasonal influenza vaccine, influenza A (H3N2), A (H1N1), and influenza B strains or their HA proteins are included, with the vaccine seed viruses replaced periodically to try to closely match the antigenicity of the virus currently circulating in the public [1,110]. The vaccines that include the two IAV strains and one of the influenza B strains are called trivalent vaccines. Due to issues with the influenza B strain in the vaccine not corresponding to the circulating strain, quadrivalent vaccines that contain components of both influenza B strains were designed. Although there has been concern over the safety and efficacy of quadrivalent vaccines compared to trivalent vaccines. A study investigated this issue by examining the antibody responses before and after immunization with either the trivalent or quadrivalent influenza vaccines, as well as the seroprotection, seroconversion, or adverse effects following the vaccination. It was observed that both vaccine platforms provided seroprotection and seroconversion and had similar adverse effects. Both vaccine platforms also met the requirements of the Committee for Human Medicinal Products for influenza vaccines [114]. Currently both vaccine platforms are used annually; however, there are different recommendations for different groups of people (e.g., recommendations based on age group) [115].

The most widely used of the three vaccine platforms are inactivated vaccines, which include whole-virion, split-virion, and subunit vaccines, in the order of the complexity of the viral component used. Immunization with inactivated vaccines can begin at 6–12 months of age [1], with the need for an annual booster. Live attenuated vaccines cause a weakened infection and can elicit both immunoglobulin (Ig)A in the upper respiratory tract and IgG in tissues and serum. Live viruses have to replicate to induce immunity, and their rate of replication is affected by the recency of a previous infection with a related strain in the host [116]. These vaccines mimic natural infection and usually induce robust immunity, but are not recommended for children younger than two years old, pregnant females, or immunocompromised people due to concerns of the state of their immune systems [1,116]. Finally, recombinant HA vaccines depend on a protein expression system using insect cells and baculovirus [117]. They have a similar mode of action to inactivated vaccines but are faster to manufacture and more scalable in production [1,113].

A number of manufacturers currently make and distribute influenza vaccines, including Sanofi (Fluzone), GlaxoSmithKline (Fluarix), Seqirus (Fluad), and MedImmune (FluMist). According to the Centers for Disease Control and Prevention (CDC), the seasonal influenza vaccine effectiveness has varied yearly from 2009 to 2020 with the lowest and highest being 19% and 60%, respectively [115]. Surprisingly, there is no upward trend in vaccine effectiveness in this 10-year timeframe. Several factors could be contributing to the varied and unpredictable vaccine effectiveness from year to year, including mismatching of the strains used in the vaccines from the circulating strain, unpredictable antigenic drift or shift that generate new circulating strains that deviates from the vaccine strains, and random cross-species transmission. In an older study by Osterholm et al., an extensive literature search led to the identification of 31 statistically and scientifically rigorous studies, which showed the pooled efficacy of trivalent inactivated vaccines to be 59% in adults aged 18–65 years (no data for other age groups), and that of live attenuated influenza vaccine (LAIV) to be 83% in children between 6 months and 7 years old [118].

mRNA vaccines represent a new class of technology based on messenger RNA, and mRNA vaccines targeting the spike protein of severe acute respiratory syndrome coronavirus 2 were the first widely used mRNA vaccines in human. The pandemic that was declared for the coronavirus disease that emerged in 2019 (COVID-19) and the urgency for a fast, scalable, and low-cost vaccine brought this class of vaccines to the front line.

One of the main features that makes mRNA vaccines an intriguing technology for control and prevention of IAV (as an RNA virus with high mutation rate) is the accurate yet flexible antigen design [119,120]. Antigenic drift and antigenic shift result in new IAV variants that can promote evasion from previous vaccine-induced immunity. mRNA vaccine technology can facilitate easier stockpiling where unformulated mRNA or low-volume libraries of plasmid can be stored for many years, and when required, this unformulated RNA can be prepared quickly for urgent uses. In addition, mRNA vaccines for influenza prevent mutation and, therefore, antigenic drift, during the process of virus replication in embryonated eggs [121]. Developing mRNA vaccines does not need pathogen growth and is a completely pathogen-free and non-infectious process [122]. Among currently used influenza vaccines, most of them are manufactured based on chicken eggs or cell substrates. Normally, this process is time consuming and depends on the accessibility of adequate pathogen-free embryonated eggs. Approximately six months is required to produce a first vaccine series and protect the highest risk subpopulations to prevent outbreaks and epidemics. This incompatibility between the pace of vaccine production and epidemic growth highlights the necessity of an alternative vaccine platform that that can be manufactured faster than conventional vaccines [123].

Immunogenicity of an unmodified mRNA vaccine encoding several influenza antigens, proved to be comparable to conventional inactivated vaccines [124], and induced a reasonably effective antibody-mediated immune response [125]. High antibody titers have been demonstrated in a human phase 1 clinical study in individuals who received an mRNA-based influenza vaccine [120,123]. The current COVID-19 mRNA vaccines have demonstrated the potential production speed of influenza mRNA vaccines. However, since this technology is new and was rolled out so quickly for COVID-19, extra vigilance should be practiced before widespread application in the context of influenza by conducting extensive safety, pharmacokinetic, and biodistribution analyses. Since mRNA vaccines cause transfected cells to transiently manufacture the target antigen(s), the implications of the immune system attacking some self-cells needs to be investigated very closely.

## 7. Original Antigenic Sin and Influenza

One of the barriers in developing a universal influenza virus vaccine is a phenomenon known as “original antigenic sin” (OAS). OAS was first described by Thomas Francis in the 1960s and refers to the concept that an individual’s first encounter with an influenza virus results in an immunological imprint. This imprinting governs Ab responses during subsequent influenza virus infections [126]. In general, Ab responses to influenza viruses are highly cross-reactive. However, OAS causes biased production of Abs against previously encountered epitopes rather than development of immunity to new epitopes [127]. This poses an issue when developing vaccines for influenza viruses, which rapidly and frequently mutate.

HA is a membrane protein on influenza viruses that consists of a globular head that differs substantially between strains, and a stalk domain that has more conserved epitopes [128]. Arevalo et al. investigated OAS priming of the HA stalk Abs in ferrets and humans and suggested that individuals exposed in childhood to H1N1 or H3N2 may have strong immunological memory against group 1 HA stalks or group 2 HA stalks, respectively [128]. Heterosubtypic infections with viruses of a different antigenic group than the viruses encountered in childhood could lead to recognition of HA stalk and production of Abs that fail to bind and protect against the boosting antigen [128]. An additional study by Meade et al. showed similar findings of back-boosting in a Nicaraguan household transmission study. The participants were assessed following infection with H1N1 [129]. They found that children under the age of six had a relatively narrow response to H1 HAs that are closely related to the HA strain that caused infection and did not induce cross-reactive Abs. In contrast, adults had much broader responses, including a boost in Abs to various seasonal group 1 subtype HAs in an OAS-like fashion [129]. These finding demonstrated that immunological imprinting has long-term effects on subsequent immune responses following vaccination or infection, which could be taken advantage of in vaccine design, and represents an opportunity to optimize peoples’ immune responses.

Another surface protein that is under investigation for OAS patterns is NA. Rajendran et al. investigated the immune responses to a panel of N1, N2, and influenza B virus NAs in different age groups and observed similar response patterns as those to HAs. The NA-specific Ab titers increased with age and were generally highest against strains that circulated during the individual’s childhood [130]. Adults and elderly people had high titers of anti-influenza B virus NA Abs, while children were almost non-reactive, possibly because they were not yet exposed to that strain of virus [130]. Similar results were also seen by Mendez-Legaza et al., which looked at NA-specific Abs following various H1N1 infections. The study’s participants exhibited different Ab responses that corresponded to viruses that likely primed their immune system upon the first infection [131] and the observations provide further evidence of OAS patterns in response to NA.

In summary, numerous studies have contributed to the understanding of OAS. Interestingly, it has been proposed that antigenic sin can be turned into antigenic ‘blessing’ through orchestration of the first encounter with influenza virus via a vaccine that delivers multiple strains’ epitopes simultaneously [132]. This would produce a diverse immune response and broad protection to subsequent influenza virus infections. As well, considering recent findings, it would be crucial to have these vaccines include epitopes that induce Ab responses to both HA and NA to overcome OAS. Further research is required to investigate if other influenza virus antigens can also cause sub-par immune responses against novel strains due to OAS and include those into the development of a universal influenza virus vaccine.

## 8. Naturally Acquired Immunity to Influenza Viruses

In the host, IAV will first face the respiratory system’s defense mechanisms including antimicrobial peptides and collectins [133]. Surfactant protein-A, -B, and mannose binding lectin are all a part of the collectin family and are pathogen recognition receptors (PRRs) that assist in IAV clearance and the attenuation of inflammation caused by IAV infection [134]. The complement lectin, L-ficolin, has been observed to bind to HA and NA of IAV and can protect hosts from IAV infection in murine models [135]. HA on IAV binds sialic acid on surfactant protein-A, and results in virus neutralization and has been demonstrated to assist in the clearance of IAV infection and reduction of pulmonary inflammation in murine models [136]. When the host’s lungs are exposed to IAV, innate leukocytes such as neutrophils, produce various antimicrobial peptides, such as human cathelicidin LL-37 [137], human neutrophil peptide-1, and human neutrophil peptide-2, which neutralize IAV [138]. Moreover, neutrophils produce human neutrophil peptide-1, which can inhibit protein kinase C (PKC) [139]. IAV replication in the host requires the hijacking of host human ribonucleoprotein complexes, which are regulated by the PKC family [140]. PKCβII, an isoform of PKC, is necessary for IAV infection and replication in host cells [139]. It has been observed that when PKCβII activity is inhibited, IAV infection is obstructed [141].

Reaching the mucosa of the host, sialic acid α2,6-galactose sialyloligosaccharide linkages of the epithelial cells in the respiratory system are the preferred target of influenza viruses [142]. The virus will fuse to the host cell, allowing it to enter the cell in an endosome. Endosomes have a low pH which allows for the virus to uptake protons via the M2 channel and results in the uncoating of the virus and release of the virus’s ribonucleoproteins [143]. IAV can be detected by host cells by multiple PRRs. Toll-like receptors (TLRs) are types of PRRs that exist on the surface of cells and in endosomes. As such, IAV can be recognized by TLRs in the endosomes. TLR3 [144,145] and TLR7 [146] can both recognize the RNA of IAV and initiate a signaling cascade that involve various components. Both TLR pathways result in the activation of transcription factors that promote the expression of type I and type III IFNs [142].

Another PRR for IAV detection is an RNA helicase called retinoic acid inducible gene 1 (RIG-1) that can recognize the viral RNA of IAV in the cytosol of hosT-cells [147]. This initiates a signaling pathway that involves the activation of mitochondrial antiviral signaling protein and results in the promotion of pro-inflammatory and antiviral activity [147,148,149]. The RIG-1 and TLR pathways both result in the production of IFN responses [143,146,148,149,150,151,152,153].

IAVs also have protective mechanisms, including methods to reduce IFN signaling [151]. IAV has also been observed to activate another family of PRRs called nucleotide oligomerization domain (NOD)-like receptors (NLRs), specifically NOD-like receptor family pyrin domain containing 3 (NLRP3) [154,155]. IAV has been demonstrated to activate NLRP3 inflammasomes, which have been observed to be essential in proinflammatory cytokine productions during IAV infection. The promotion of inflammation in the respiratory tract by NLRP3 was observed to have a protective effect in murine models of IAV infection [154].

IFNs have been demonstrated to be necessary for the activation of inflammasomes and production of various pro-inflammatory cytokines including, interleukin (IL)-1β and IL-18, that have protective roles against IAV infection [156]. Some of the main cytokines produced during IAV infection are TNFα, IL-6, IL-1β, and IFNs [153]. Cytokine responses during IAV infection are important in regulating inflammation, promoting anti-viral responses, and recruiting and activating leukocytes [153].

Host cellular mediators during IAV infection include respiratory epithelial cells, pulmonary endothelial cells, and leukocytes. Leukocytes involved in the protection of IAV infection include natural killer (NK) cells, neutrophils, dendritic cells, and alveolar macrophages [143]. Alveolar macrophages and monocytes are recruited by C-C motif chemokine ligand 2 produced by epithelial cells that have been infected with IAV. Along with other functions, these macrophages and monocytes will phagocytose virus-infected apoptotic cells, thereby limiting viral spread [157]. Neutrophils also assist the host defense against IAV via phagocytosis, clearance of debris, killing of virus-infected cells, release of granules, and recruiting other leukocytes [133,158]. Conversely, neutrophils have also been associated with promoting inflammation, induction of lung damage, and poor patient outcomes [159]. Increased expression of neutrophil activation in hosts was associated with increased disease severity and could be used as a predictive marker for patient outcomes [160].

Dendritic cells can detect IAV through various pathways, for instance plasmacytoid dendritic cells can detect IAV via TLR7 [146] and conventional dendritic cells can recognize IAV through RIG-1 [144,146]. Dendritic cells are potent antigen-presenting cells with crucial roles in the communication between innate and adaptive immunity, making them key innate leukocytes [133]. Dendritic cells in the respiratory system can produce inflammatory cytokines and capture IAV antigens for education of adaptive cell-mediated immune responses. IFNs can stimulate the maturation of dendritic cells, such as those residing in the respiratory tract. Mature dendritic cells that have acquired IAV antigens can migrate to lymph nodes and prime and activate T-cell responses against IAV [161]. This includes promoting virus-specific cytotoxic T-cell responses that can kill infected hosT-cells [162,163]. Dendritic cells also help support antibody responses by presentation of antigens to B-cells and in generation of plasma cells from B-cells, and it has been observed that in the absence of dendritic cells, antibody responses against IAV are compromised [164].

Influenza viruses in a host will initiate many different signaling pathways of the host defense response. Despite the plethora of defense mechanisms against influenza, influenza viruses manage to adapt and develop protective mechanisms that allow for replication and survival. Qualitative aspects of host responses vary from person to person depending on genetic factors and immunological imprints [160,165]. Further complicating influenza infection, is the fact that host responses can even inflict self-harm due to losses in regulation and/or hyperactivation that can cause various injuries [163,166,167]. Influenza infections involve many complex factors and pathways that should be considered in vaccine design and taken advantage of in order to promote sterilizing immunity.

### Type 1 Versus Type 2 Immunity in Influenza Vaccination

The immune system can elicit qualitatively different responses to optimally respond to distinct species of pathogens. It is important to design vaccines that capitalize on these mechanisms to produce a tailored and maximally protective immune response. In 1986, Mosmann et al. [168] first described the existence of two major functionally different subsets of CD4+ T helper (Th) cells, distinguishable by the cytokines they produce and the different regulatory and effector functions they mediate in response to invading pathogens. CD4+ Th1 cells are primarily associated with the induction of pro-inflammatory responses, increased phagocytic activity, and cytotoxic CD8+ T-cell activation [168,169]. Conversely, CD4+ Th2 cells primarily regulate B-cell activation and Ab responses [168,169]. The T helper cell type hypothesis subsequently gave birth to the concept of type 1 versus type 2 immunity, which applies to many leukocytes exhibiting both type 1 and 2 phenotypes. Type 1 responses function primarily to protect against intracellular pathogens, such as viruses, through cytokine production, upregulation of innate leukocyte phagocytic and antigen-presentation activities, and induction of cytotoxic T-cell expansion essential in the killing of virally-infected cells and induction of CD4+ and CD8+ memory T-cell responses [170]. Conversely, type 2 immune responses function to protect against extracellular pathogens, such as parasites, and involves the initiation of B-cell class switching, and the production of Abs [169].

The polarization of type 1 versus type 2 immunity is of particular importance for consideration in influenza virus vaccine development, given that the most effective responses against intracellular organisms like influenza viruses are type 1 in nature [170,171]. Less severe cases of natural infection with influenza viruses have been associated with the accelerated induction of Th1 responses, while Th2-biased responses have been strongly associated with enhancement of lung pathology and disease progression due to reduced viral clearance [108,166,167,172]. Thus, vaccines formulated to tilt the balance in favor of type 1 immunity are vital for eliciting more effective and rapid responses upon influenza re-infection, particularly in scenarios of incomplete antibody-mediated protection. It is, therefore, pertinent that the vaccine platform elicits the appropriate initial response, in order to generate a cytokine microenvironment conducive to promotion of type 1 polarization. Several factors influence the polarization of type 1 versus type 2 immunity, the most notable of which include the choice of vaccine platform and the subsequent relative immunogenic strength of the vaccine-derived viral antigens, the local cytokine milieu stimulated upon administration, the dose and route of administration, the antigens of choice, and subsequently, the type of antigen-presenting cell stimulating the T-cell [167,168,169,170,173,174].

The vaccine platform will influence the type of immunity the host will respond with and can affect the efficacy of the vaccine. For instance, inactivated or subunit vaccines may not have the capacity to enter the host cell as the virus would during natural infection due to factors such as inactivation or an absence of components that allow entry into the cell. The host immune system may incorrectly interpret it as an extracellular pathogen and elicit type 2 immunity rather than as an intracellular pathogen and elicit type 1 immunity. Major pathways of host detection of influenza virus first require attachment and fusion of the virus to the host cell before recognition of its viral RNA can occur [107]. If the vaccine is designed in such a way that it is recognized as an extracellular pathogen, or does not mimic influenza infection sufficiently, this will result in improper interpretation and sub-optimal host responses, which can be imprinted into the host’s immunological response memory.

Currently available seasonal inactivated influenza vaccines have been observed to reduce efficiency in inducing type 1 immune responses, potentially contributing to their limited efficacy and breadth of reactivity against diverse influenza virus strains [175]. The inactivated influenza vaccines are also poor stimulators of heterosubtypic cell-mediated immunity needed to prevent the serious complications of influenza infection, which would otherwise be elicited by natural influenza virus infections. Live-attenuated influenza vaccine strains were observed to induce superior protection against influenza infection in children and adults with pre-existing immunity, which researchers attributed to the ability of the LAIV to stimulate potent Th1 cell responses [175,176,177]. While both platforms have been observed over multiple studies to induce similar viral hemagglutination inhibition (HAI) Ab responses, only LAIVs induce significant increases in T-cell responses. These lines of evidence coupled with the continuous emergence of antigenic drift variants of seasonal influenza viruses suggests that considerable attention should be paid to enhancing influenza vaccine platforms to induce broader protective immunity, rather than a bias in induction of strain-specific Abs to the viral HA. Since this could be afforded by vaccinations biased towards stimulating the Th1 cascade [178,179], there is consequently a need to investigate how Th1 cell-mediated immune responses can be enhanced in the design of new influenza vaccines. Multiple mechanisms to mediate vaccine-induced type 1 polarization have been described, including the use of IFN-γ and IL-12 as vaccine adjuvants or the inclusion of genes coding for IFN-γ or IL-12 in the vaccine platform. Reports have been published of phase I and II clinical trials utilizing platforms biased to polarize patients’ immune responses toward the appropriate phenotype, with promising results thus far [180,181]. The inclusion of molecules capable of disrupting or inducing transcription factors regulating Th1/Th2 polarization may provide an additional set of clinical tools in the influenza vaccine platform toolbox.

While the stimulation of Th1 responses can inhibit disease progression through more efficient clearance of the virus, vaccines targeted towards heightening Th1 responses generally require the use of conserved epitopes that can be recognized by all major histocompatibility complex subclasses. Given the extensive variability of epitope recognition across individuals, the feasibility of generating vaccines with this ability is limited. Despite type 1 responses being pertinent to and favorable for optimal protection against influenza infection, paradoxically, clinicians traditionally measure vaccine-induced protective immunity by following Ab titers [182]. While the Ab response to influenza vaccination may indeed be a good correlate of protection, high Ab titers can be consistent with either type 1 or type 2 immunity depending on the subtypes of Ab present and, therefore, may not be predictive of the degree of protection against severe influenza illness. One improvement in monitoring influenza vaccine rollouts would be to expand Ab testing to include analysis of Ab subtypes associated with type 1 versus type 2 immunity. Clinically, there are no rapid cell-mediated immunological assays; thus, it is difficult to know to what degree a given vaccine elicits type 1 versus type 2 immunity in a patient. Only after directly analyzing cytokine patterns elicited following vaccination, or concurrently studying humoral and cell-mediated responses can one comment on the relative effects of a vaccine in terms of the type 1 versus type 2 paradigm. Future studies of influenza vaccination focused on expanding the criteria used to categorize vaccine responses to include more than just HA Ab titers will be informative for confirming correlates of protection affiliated with vaccine efficacy.

## 9. Trained Immunity and Influenza

Trained immunity is a recently discovered phenomenon that occurs in innate leukocytes, when they are exposed to certain pathogenic stimuli, and develop a non-specific immunological memory through epigenetic, functional, and metabolic reprogramming [183,184,185,186]. Clinical studies indicate that trained immunity can be used to enhance immune responses against infections and improve vaccine efficacy in adults [183]. However, it is unclear what promotes or restricts vaccine effectiveness. As mechanisms underlying trained immunity are better understood, they can be exploited in the design of new therapies and vaccines that combine activation of classical adaptive immune memory and trained immunity, which will be an important area of future research [187].

The process of trained immunity following infection and vaccination involves members of the innate immune system such as monocytes, dendritic cells, and NK cells. These cell types exhibit increased reactivity to a second infection that may be the same or a different pathogen [183]. For instance, beta-glucans are a group of polysaccharides found naturally in the cell walls of bacteria and fungi, which are high in biologically active polysaccharides and are recognized by PRRs on leukocytes. In mouse models, these substances were observed to improve the immune response of the host by stimulating and improving the functions of innate leukocytes, and resulted in protection against bacterial infections that cause peritonitis, enteritis, and pneumonia [188].

Bacillus Calmette-Guérin (BCG) vaccination protects against tuberculosis, but it also protects against viral diseases, including respiratory syncytial virus, human papilloma virus, and herpes simplex virus [187]. In eliciting protective responses against *Staphylococcus aureus* or *Candida albicans*, BCG vaccination induces trained immunity in monocytes and NK cells, resulting in increased expression of activation markers and development of pro-inflammatory cytokines [189]. Employing flow cytometry technology, it was demonstrated that BCG vaccination protects mice from IAV infection by promoting the induction of memory T-cell responses [190]. BCG vaccination was shown to reduce influenza virus titers, and virus-induced inflammation and lung injury compared to a control group [187]. Delivering the BCG vaccine to the lungs greatly improves efferocytosis by alveolar phagocytes, and this increased efferocytosis protects mice from fatal influenza-mediated pneumonia by supporting the maintenance of pulmonary homeostasis [191].

BCG vaccination induces trained immunity, which results in a more effective cytokine response, including cytokines associated with antiviral responses such as IL-1, tumor necrosis factor, and IFNs. These cytokines are present in high circulating concentrations in patients with the severe acute respiratory syndrome-coronavirus-2 (SARS-CoV-2) and may contribute to acute respiratory distress syndrome (ARDS) [186]. When the BCG vaccine is given before influenza vaccination, functional antibody responses to the 2009 pandemic influenza A (H1N1) vaccine strain were significantly increased in concentration and their induction was accelerated. These findings could have implications for vaccination strategy development and may contribute to increased vaccination efficacy. However, a study performed by de Bree et al. observed that the BCG vaccine did not promote protection of mice against avian H7N9 influenza and did not affect histopathological damage, inflammation, or viral replication. No significant differences in survival, weight loss, or pulmonary disease was demonstrated between the groups that had previously been immunized with BCG and the control groups that had not [192]. Therefore, although trained immunity from BCG vaccination is a promising strategy to examine against influenza infection, its benefits may be strain-dependent, and further investigations are needed.

A current relevant topic similar to influenza viruses is SARS-CoV-2, which is the causative agent of COVID-19. As a result, there has been a great deal of interest in SARS-CoV-2 and its potential similarities and relationships with influenza viruses. The aim of several retrospective observational studies was to see if there was a link between trivalent influenza vaccination and COVID-19 mortality and serious clinical outcomes in hospitalized patients. Patients with at least two of the following symptoms were assigned a diagnosis of respiratory infection: fever, chills, sore throat, headache, cough, or loss of smell or taste. This retrospective observational study demonstrated that patients with COVID-19 who had recently received an inactivated influenza vaccine had substantially improved health outcomes compared to non-vaccinated patients [193].

Several studies have proposed that influenza virus infection can induce a trained immunity response that improves cytokine responses to SARS-CoV-2. In vitro experiments investigating human leukocyte responses to SARS-CoV-2 revealed that responses can be induced by an inactivated influenza vaccine, which may result in relative protection against COVID-19 [194]. Influenza vaccination has been linked to fewer positive COVID-19 tests and better clinical outcomes, which suggests it could be providing a form of protection, possibly through mechanisms of trained immunity [195].

A study done by Priya et al. using an established in vitro model of trained immunity, demonstrated that the inactivated influenza vaccine can induce a trained immunity response, including an improvement in cytokine responses after stimulation of human leukocytes with SARS-CoV-2 [194]. During the first six months, memory NK cells with intracellular NKp46 expression were induced by influenza vaccines. Increasing NKp46 was positively associated with increased IFN-γ production, which could react quickly to viral restimulation. This finding indicates that the ability of the NK cell memory-like response to provide strong immunity against a variety of influenza virus subtypes may be an advantage of trained immunity [196]. More research is needed to determine the role of trained immunity in influenza vaccinations and COVID-19 infection.

LAIVs have previously been linked to increased pro-inflammatory cytokine production and an effect on both innate and adaptive immune responses. Cold-adapted LAIV, which is more immunogenic in anatomical locations below 37 °C, induces local innate immune responses that provide a broad range of antiviral immunity. Cold-adapted LAIVs can provide short-term, non-specific defense against genetically unrelated respiratory pathogens. The vaccine triggered an almost immediate release of cytokines and leukocyte infiltration into the respiratory tract, reducing the immune disruption caused by respiratory syncytial virus infection [197], illustrating how vaccine platforms can influence the trained immunity responses that are elicited. Further investigation into trained immunity responses following different vaccine platforms would be informative for future vaccine designs and could be useful for development of vaccines that can be catered for specific requirements.

Trained immunity-based vaccines are a relatively new idea that involve the priming and education of innate leukocytes. The stimulation of innate leukocytes through their PRRs causes epigenetic changes that will affect the subsequent innate immune responses. Therefore, this concept can be capitalized on as an approach to design vaccines that train the innate immune system. In contrast to traditional vaccines that aim to develop immune responses against specific antigens, trained immunity-based vaccines would develop a more broad and non-specific immunity in the host [198].

There are investigations into the development and use of prophylactic vaccines that focus on trained immunity for individuals that are immunocompromised and would benefit from an enhanced broad immune protection such as individuals with immune disorders who are more prone to developing infections [199]. A study by Guevara-Hoyer et al., has demonstrated the possible benefit of this concept. They observed a positive effect in patients with common variable immunodeficiency from an adjuvant trained immunity-based vaccination, wherein they had reduced upper respiratory infections and decreased medical attention requirements and expenses [199]. This demonstrates the exciting potential to generate trained immunity-based vaccines that could have useful applications in the design of influenza vaccines to enhance broad cross-reactive immunity, especially for immunocompromised individuals.

Trained immunity is a relatively new concept in the field of immunology and could be used as a tool to improve influenza vaccination efficacy. Further investigation into the roles of trained immunity in the immune system and responses to foreign pathogens such as influenza virus will allow for a better understanding of the molecular mechanisms that promote innate memory-like responses. Current vaccine strategies primarily focus on adaptive immunity. However, innate immunity may play more important roles in vaccination than previously considered. Vaccines designed to target and educate both innate and adaptive immunity may be the key to unleashing the full potential of influenza vaccines and lead to a new generation of vaccines.

## 10. Immunological Immaturity and Influenza Vaccines

Infection with influenza viruses presents a serious health concern for immunologically immature individuals, including fetuses, neonates, and young children, which are at a higher risk of infection and are more vulnerable to serious consequences than adults [200]. This significant burden results from limitations in both the innate and adaptive immune system [201]. The infant immune system is tasked with adapting to its new environment, learning to accommodate commensal microbiota, and neutralizing harmful antigens [201]. This rapid change results in phenotypic differences in immunological characteristics compared to adults, including antigen presenting cells and immunoglobulins [201]. The first exposure to a virus is an important immunological event as it can define the immune response to subsequent viral infections [202]. It is very important that the first exposure to an influenza vaccine is an effective one and understanding these imprinting events will better inform future vaccine development.

As of now, inactivated vaccines are the only vaccine type authorized for use in children under two years of age [203]. While LAIV have demonstrated superior efficacy to inactivated vaccines, they increase the risk of the obstruction of airways in patients under 12 months of age [204]. The pathogenesis of this wheezing is unknown and until studies are conducted to elucidate the underlying mechanisms contributing to this side-effects, LAIV will not be recommended for children under 12 months [204]. Unfortunately, immune responses to the standard inactive vaccines have demonstrated inadequate effectiveness in young children [205]. Strategies to enhance the immunological education and limit the negative side effects of influenza vaccines must be identified to maximally protect this high-risk population.

Due to the mismatch between the B lineage influenza virus in circulation and the one in the seasonal vaccine, trivalent vaccines cannot provide guaranteed protection against influenza B, to which children are especially vulnerable [206]. Thus, quadrivalent vaccines which include both influenza B viruses were developed where an immune response is elicited against four different antigens [206]. These quadrivalent vaccine platforms have been extensively studied and approved in populations over the age of three years old. However, research was lacking in the safety and immunogenicity of these vaccines in children under three. With this high-risk population in mind, Pepin, and colleagues conducted a phase III clinical trial and demonstrated that these vaccines were both effective and safe when compared to the currently approved trivalent vaccine in participants aged 6–35 months [207].

Adjuvants are used in vaccines to induce more robust immune responses [208]. The adjuvant known as MF59 stimulates antigen-presenting cells and promotes T-cell activation and B-cell expansion [208]. The broadened immune response to adjuvanted vaccines may more closely mimic a natural infection and since first exposures are critical, this could provide long-term benefits [209]. MF59-adjuvanted trivalent and quadrivalent vaccines have been shown to be both safe and highly immunogenic when compared to their non-adjuvanted counterparts [210,211]. To properly prime and activate the immune system, two doses are recommended for non-adjuvanted vaccines in an infant’s first influenza season [203]. It was demonstrated that one dose of an adjuvanted inactive vaccine provides a better Ab response than two doses of a non-adjuvanted inactive vaccine [209]. This may provide a more logistically effective dosing regimen than what is currently recommended. The long-term benefits of priming have yet to be elucidated, and research into the continued protection from these adjuvants is required.

Efforts to protect children in their first six months of life through vaccination have been unsuccessful. The safety and efficacy of any type of influenza vaccine has not been shown in infants. Influenza vaccination during pregnancy and the subsequent passive immunity is an important strategy for the protection of infants in their first months of life [212,213]. By vaccinating mothers, infants have a substantially decreased risk of influenza and influenza-related hospitalizations [212,213]. The efficacy of these vaccines is highly dependent on timing [214]. The timing in which the mother receives the vaccine, the time between vaccination and birth, and the timing of influenza circulation can limit protection for the first two to three months of life [214]. This demonstrates the need for highly immunogenic vaccines to increase the concentration of Abs transferred from mother to child and extend the period of protection. As of yet, no vaccine that fills this need has been identified.

## 11. Immunosenescence, Influenza, and Influenza Vaccination

Elderly individuals are a key demographic for protecting against influenza as they are disproportionately at risk for higher rates of influenza-related severe diseases, hospitalization, and death [215]. Despite their importance, the efficacy of influenza vaccines are markedly lower in elderly (≥65 years of age) individuals, with efficacy rates ranging from 17–53% [216]. This increased susceptibility to influenza and reduced vaccine efficacy is rooted in poor immunological functioning due to progressive dysregulation of the immune system, termed immunosenescence. Due to reduced functioning in both innate and adaptive leukocyte activities, the issues with current influenza vaccines are exacerbated in the elderly. As such, a greater focus needs to be given to optimizing influenza vaccines for the elderly.

The combination of immunosenescence and immunological aging in elderly individuals leads to a progressive dysregulation of the immune system that affects all leukocyte populations. Detailed changes occurring in innate and adaptive leukocytes populations as a result of immunosenescence has been extensively reviewed by Allen et al. [217], and is briefly reviewed here. Aged phagocytes, such as neutrophils, monocytes, macrophages, and dendritic cells have reduced phagocytic ability, compromising the ability to kill and engulf pathogens [218,219,220]. Elderly individuals also have a reduced capacity to combat viral and malignant threats due to dysfunctional migration and cytotoxicity of NK cells [221]. Reduced phagocytosis and NK cell cytotoxicity likely results in a reduction in the number of viral peptides produced and acquired during infection or vaccination contributing to reduced magnitude and breadth of adaptive responses. A variety of age-related defects in dendritic cells likely also plays a major role in reduced immunity in the elderly. Dendritic cells have decreased ability to become activated, have poor antigen uptake and presentation, and reduced expression of co-stimulatory molecules, all of which may contribute to reduced activation of adaptive responses [222,223].

The functionality of aged leukocytes of the adaptive immune system are affected at multiple levels. First, age-related involution of bone marrow and the thymus reduces the production of naïve B and T-cells, greatly reducing the overall diversity of the B and T-cell receptor repertoires [224,225]. As a result, aged immune systems have reduced capacity to generate de novo immune responses against novel epitopes. With reduced pools of naïve B and T-cells, aged immune systems compensate by relying more heavily on memory responses [226]. The effectiveness of current influenza vaccines primarily relies on the ability to generate de novo Ab responses against antigenically shifted epitopes within the influenza HA protein. Therefore, current influenza vaccine strategies are poorly designed for efficacy within elderly populations. In addition to reduced pools of naïve lymphocytes, there are numerous phenotypic differences in the B and T-cells available. For example, B-cells of aged immune systems exhibit a reduced ability to undergo somatic hypermutation and class switch recombination [227]. The Abs that are produced demonstrate reduced affinity maturation and suboptimal effector functions, greatly reducing their ability to neutralize viruses [228]. Combined with restricted clonal expansion, B-cell responses are severely hindered in elderly individuals [229]. Reliance of current influenza strategies on antibody-mediated immunity against antigenically shifting HA epitopes is a poor strategy for protecting high-risk elderly individuals.

Similar to B-cells, T-cells of elderly individuals exhibit numerous age- and immunosenescence-related defects. Interestingly, with increased age the ratio of CD4+ to CD8+ T-cells increases, which is accompanied by an increase in differentiated memory T-cells [230]. Within this larger population of CD4+ T-cells there are numerous defects such as reduced production of IL-2, reduced capacity for clonal expansion, and altered differentiation upon antigen stimulation [231]. Elderly individuals fail to produce increased numbers of activated T follicular helper cells, reducing the ability to produce effective Ab responses [232]. Similarly, compromised CD4+ T-cell function can also negatively affect the generation of cytotoxic T-lymphocyte responses. Lastly, CD8+ T-cells have lower cell surface expression of CD28 and reduced cytolytic abilities, which represents a significant disadvantage in immune protection [233]. Dysregulation of innate and adaptive leukocyte populations positions elderly individuals at a severe disadvantage in properly controlling influenza infections and optimally responding to vaccination. Therefore, there is a critical need for novel strategies to overcome these immunological defects.

Since current influenza vaccines focus on generating HA-specific Abs, there is a significant amount of literature characterizing B-cell responses in the elderly. Elderly individuals have consistently been shown to reduce vaccine-induced Ab responses characterized by decreased IgA and IgG concentrations, delays in achieving peak Ab titers, and rapid decline of Abs [234]. One study investigating the epitope specificity of Abs generated from influenza vaccination demonstrated that the magnitude of polyclonal antibody responses following vaccination did not significantly differ between elderly and young volunteers [234]. Upon closer examination of epitope specificity, the authors demonstrated that the amount of HAI-positive mAbs was substantially reduced in the elderly, 33% versus 72% [234]. Rather than targeting the HA protein, the majority of the vaccination-induced Abs targeted rare epitopes in other influenza proteins, such as NA, NP, and others. Therefore, while the total magnitude of Ab responses was similar between the two groups in this study, the overall magnitude of the anti-HAI response was greatly diminished in elderly individuals.

Another shortfall of current influenza vaccines is their inability to generate long lasting Ab responses in the elderly population. Vaccination-induced influenza virus-specific Ab responses have been shown to last less than one year in the elderly, with some studies having demonstrated insufficient HAI Ab titers as early as 120 days (four months) post-vaccination [235,236]. As a result, some members of the elderly population are insufficiently protected for the influenza season. Similarly, vaccine induced CD4+ T-cell responses have been shown to be shorter lived in elderly individuals [237].

With increasing age and the development of comorbidities, aged individuals often require the long-term use of medication to manage their health. A recent study by Agarwal et al. demonstrated that elderly individuals on long-term metformin therapy had significantly lower IgA, IgG, and IgM Ab responses following influenza vaccination [238]. Surprisingly, 28 days post-immunization the virus neutralizing activity of patient sera was 37-fold lower for metformin users compared to non-users. Reduced virus neutralizing activity of metformin users was demonstrated at all later study time points. Interestingly, metformin users had a 25% higher proportion of CD8+ T-cells at baseline, which increased to 41% following vaccination. This study did not determine the protective efficacy against influenza infection, thus, it is unclear if the enhanced CD8+ T-cell responses in metformin users would be beneficial or detrimental. The results of this study underline the importance of considering the effect of medication on influenza vaccine efficacy and that other unexpected factors may affect the immune responsivity of elderly individuals.

Despite vaccination representing the best strategy for protection against influenza, influenza vaccines remain poorly effective in elderly individuals, with efficacy ranging from 17–53% [216]. Therefore, there is a need for novel strategies capable of overcoming barriers associated with immunosenescence to provide protection against influenza to the elderly population. Optimization of vaccine immunogenicity requires a multipronged approach, including optimization of current vaccination protocols, trained immunity-based vaccines, and social interventions.

## 12. Immunocompromised Individuals

Immunocompromised individuals are at an increased risk of serious complications from infections with influenza viruses [239]. Due to the weakened nature of the immune system, immunocompromised populations are recommended to get influenza vaccines with inactivated virus [240]. Compromised humoral and cell-mediated responses will result in an impaired ability to produce Abs necessary to prevent infection and a hindered ability to clear viral particles and limit the spread and repercussions of the viral infection. There is a need for increased awareness and education of the importance of receiving the annual influenza vaccination in immunocompromised individuals and healthcare workers [241,242]. Although it is highly recommended for many immunocompromised populations to get the influenza vaccine annually because of the increased risk of fatal conditions, these populations remain heavily under-vaccinated [243,244]. Immunocompromised individuals have higher rates of pneumonia and mortality from influenza and longer hospital stays than non-immunocompromised individuals [245]. Immunocompromised individuals comprise a large variety of populations including those on immunosuppressants, with human immunodeficiency virus, on steroids, taking immune-modulating agents, with cancer, with autoimmune diseases, inflammatory conditions, or transplant recipients [246]. Immunocompromised individuals are at higher risk of many health complications and disease [246]. Interestingly, it was observed that immunocompromised individuals had less detectable symptoms of influenza infection than the non-immunocompromised. This suggests they might have different clinical manifestations and there may be a need for different markers of disease since the symptoms stray from that of the general public, which could be due to different infection kinetics or pathogenesis [239].

In patients with solid organ transplants, natural infection with influenza virus was linked to increased CD4+ T-cell responses compared to vaccinated individuals who were not infected, suggesting a sub-optimal design of influenza vaccines for this demographic [247]. Transplant recipients must take immunosuppressants to prevent rejection of the transplant, which makes influenza infection a concern. Immunosuppressants are given to the recipient and function to repress their immune system from attacking the transplanted foreign material. This will also downregulate the immune responses and the host protection against influenza infection and compromise the efficacy of the influenza vaccines [247]. Transplant recipients are recommended to get the inactivated vaccine annually, but due to problems with vaccine design and the fact that their immune system may not be able to fight the infection, they are still susceptible to infection [247].

Natural infection with influenza virus in patients with transplants resulted in stronger Ab responses with a greater variety than vaccination with a split virus vaccine [248]. It has also been shown that booster vaccinations provide additional immunogenic protection in recipients of solid organ transplants [249]. A high-dose influenza vaccine conferred better immunological protection against influenza virus infection than the standard dose in patients with solid-organ transplants [250]. Cell-mediated and antibody responses to influenza following vaccination were assessed in patients with stable kidney transplants and it was observed that they had reduced T-cell and Ab responses compared to controls. Although there were no substantial side-effects observed following vaccination, the immunosuppressants the transplant recipients had to take, dampened their immunological memory and responses to influenza [251]. A study investigating the kinetics of the antibody responses following influenza vaccination in recipients of renal transplants found that there was a delay in the induction of influenza-specific Abs, as well as overall weaker responses in the transplant recipients compared to controls who had not received transplants and were not immunocompromised [252].

Obesity is a risk factor for chronic and infectious diseases with serious complications resulting from influenza infection [253]. Obesity has consequences on the immune system that compromise the ability of a host to fight influenza infection [254]. During the 2009 IAV pandemic, an increased risk for obese individuals to contract severe influenza infection and require hospitalization compared to non-obese individuals was observed, indicating obesity is a risk factor for more serious outcomes or complications of influenza infection [254,255]. Humoral responses have been observed to be negatively affected by obesity in both young and elderly populations, which result in compromised immune responses following influenza vaccination and against influenza infection [256]. Reduced CD8+ T-cell functions were apparent in obese individuals compared to those of healthy weight [257]. Diet or genetically-induced obesity in mouse models demonstrated that influenza infection has severe outcomes in obese populations compared to controls [254]. In mouse models of obesity, reduced numbers of regulatory T-cells, importantly in the bronchoalveolar fluid, have been detected, suggesting that increased lung pathologies in obese individuals with influenza infection might be due to a reduction in the ability to down-regulate overly robust immune responses [254]. Obesity has also been associated in murine models with interference of the healing processes in the lungs and can cause influenza infections to have serious and even fatal consequences [258]. Humoral responses were observed to be hindered in obese mice and humans, which may also help explain the reduced vaccine efficacy and capacity to fight influenza infection [259].

There is still doubt about the most effective and safest vaccine platform, timing of vaccination, and amount of vaccine doses in immunocompromised populations. Moreover, the degree of immunosuppression and the extent of harm the influenza infection causes can vary depending on many factors. These include the underlying condition and the treatments being given, which makes influenza vaccination more complex [245]. Although studies have shown beneficial effects from high doses and booster vaccinations, at the current moment the main recommendation for immunocompromised populations is to receive the annual inactivated influenza vaccine at the standard single dose [240]. There are different methods that are being researched to improve vaccine immunogenicity in immunocompromised individuals by addition of adjuvants, such as MF59 or increasing vaccine dosages [248,260].

### Optimization of Current Influenza Vaccines for Immunosenescence

Simple modifications to current influenza vaccines are a cost-effective, timesaving, and low-risk strategy for enhancing the efficacy of influenza vaccines. First demonstrated in 1994, increasing the dose of HA delivered was shown to enhance anti-HA and neutralizing Ab levels compared to the standard dose [261]. Since then, numerous studies have confirmed the benefit of increased HA dose in enhancing vaccine-induced Ab responses and protection against influenza [262,263]. For example, the Fluzone high-dose influenza vaccine, which contains four times the standard dose of HA antigen (60 µg versus 15 µg), has been shown to be 24% more effective in preventing influenza in elderly adults relative to the standard-dose vaccine [262,263]. It is hypothesized that increasing the amount of Ag results in greater Ag uptake and subsequent presentation by dendritic cells, leading to enhanced adaptive responses and possibly provides support for B-cell maturation in germinal centers.

Another simple strategy for enhancing influenza vaccine efficacy is the use of intradermal (ID) vaccination [264,265,266]. Holland et al. compared standard-dose ID versus intramuscular influenza vaccination in elderly individuals and demonstrated that ID vaccination provided superior seroprotection and seroconversion rates [266]. ID vaccine administration is believed to enhance vaccine immunogenicity due to the greater number of antigen-presenting cells present within the dermis, allowing for enhanced Ag presentation and subsequent immune responses. Interestingly, all currently licensed influenza vaccines are given via intramuscular injection, and it is unclear if they have been tested via ID administration. Due to its extremely cost-effective nature, we recommend that ID administration of currently licensed influenza vaccines be investigated to potentially enhance vaccine efficacy.

Multi-site vaccination is a powerful, economical strategy, which has been severely underutilized in the field of vaccinology. First pioneered in 1984 by Warrell et al., multi-site ID, and subcutaneous vaccination was demonstrated to induce rapid, high-titer Ab responses relative to conventional single injection strategies in patients [267]. We recently confirmed and extended this strategy to the field of cancer immunotherapy. Using a replication-deficient human serotype 5 adenovirus vector as a vaccine in a model of murine melanoma, we demonstrated that four intramuscular injections, with the same total vaccine dose, generated superior Ab and CD8+ T-cell responses compared to one- or two-site injection strategies [268]. The robust production of CD8+ T-cell responses using this strategy may be beneficial in the generation of universal influenza vaccines focused on generating strong influenza-specific CTL responses in the elderly. We predict that multi-site injection provides superior immune responses by maximizing the engagement of multiple secondary lymphoid tissues. Multi-site vaccination is a simple, low-cost strategy to enhance vaccine response; it should be investigated as a way to enhance vaccine responses in the elderly. The combination of multi-site injection, high Ag dose, and ID administration may prove to be an ideal strategy to maximize the efficacy of current influenza vaccines.

Earlier in this review, we discussed how some studies have demonstrated the ability of pre-immunization with the live attenuated tuberculosis BCG vaccine to enhance the immunogenicity of vaccines against numerous infectious diseases. A study investigating the effects of BCG pre-immunization on influenza vaccine efficacy in young healthy adults, demonstrated that BCG pre-immunization followed by influenza vaccination two weeks later, resulted in enhanced HI Ab responses and more rapid seroconversion [269]. While this strategy has not been tested for enhancing influenza vaccine responses in elderly populations, BCG vaccination has been shown to be safe and reduce the prevalence of acute upper respiratory tract infections in the elderly [270]. Due to an impressive track record in terms of trained immunity efficacy and safety, the BCG vaccine is a promising initial candidate to investigate the ability of pre-immunization and trained immunity to enhance the efficacy of influenza vaccines in the elderly. Further research is needed to identify optimal trained immunity-based vaccine strategies and formulations for enhancing influenza vaccine efficacy, especially in elderly populations.

## 13. The Effect of Sex on Infections with Influenza Viruses and Potential Implications for Vaccination

Sexual dimorphism of the immune system has been thoroughly documented in cases of viral, bacterial, and fungal pathogens, as well as autoimmune diseases [271]. We observed sexually dimorphic antiviral responses in murine models (Bridle and Karimi unpublished data). IAV severity is impacted by both biological sex and socioeconomic factors linked to gender [272]. Sex and gender contribute to host responses to vaccination, antivirals, and IAV infection severity [272]. The effect of sex on immune responses is affected by which life stage an individual is at [273]. Prior to puberty, males are more likely to have severe IAV infections compared to female youth. This trend reverses in adults aged 20–65 [272]. In Australia, males aged 0–14 years of age and 85+ had higher infection rates than their female counterparts [274]. Within children from Costa Rica, infections with IAV occurred more frequently in boys than girls, whereas Influenza B virus exhibited the opposite trend [275]. Both pregnancy and menopause status are relevant information that should be recorded in surveillance data and considered during clinical trial design [274].

These observed epidemiological differences in infection rates can be explained by studies that elucidate mechanistic variances in the immune system gene profile. A comprehensive research effort spanning 3672 samples from six continents determined that humans have an immunological sex-related gene expression signature in healthy adult human blood involving 144 genes [276]. Female profiles exhibited higher gene expression from their CD4+ T-cells, whereas males had elevated gene expression from myeloid cells. A generated score based on the gene expression data could be correlated with male Ab responses to influenza infection [276]. A different study analyzing the effects of e-cigarette use on live attenuated influenza virus-induced gene expression determined that significant variations existed between male and female expression profiles [277]. Genes associated with T- and B-cell adaptive immunological functions, including expression of CXCL12, CXCL13, and CCL20, IFN responses (including IFI27, IFNAR1, IFNL1, IL-18, IRF2, IRF5), and CD40- and TLR7-mediated responses to pathogens were varied, implicating differential ability to produce Abs and mount an effective memory response. Females often have a more protective anti-influenza response than their male counterparts [277].

Females have more robust Ab responses after influenza vaccination. A study that used both BALB/c and C57BL/6 strains of mice determined that female mice have higher IgG Ab titers regardless of the strain or age (three months versus 18 months) in response to trivalent inactivated split-virus influenza vaccination [278]. In an H1N1 vaccination model, female mice produced a larger quantity of Abs [279]. Antibodies obtained from female mice were more protective when transferred to naïve mice of either sex compared to Abs derived from male mice. Female B-cells expressed higher concentrations of TLR7 and deletion experiments demonstrated that a lack of TLR7 diminished sex-based differences [279]. Hence, TLR7 sexual dimorphism plays an important role in vaccine efficacy. Experiments in outbred Swiss mice examining differences between sexes in response to whole virus trivalent inactivated influenza vaccination determined that females had an elevated IgM Ab response to all three viruses and elevated IgG Abs for H1N1 [280]. There was no difference in the IgG response to either the B or H3N2 influenza viruses. These studies demonstrate that it is paramount for influenza researchers to include both male and female animals within preclinical trials. Variations in post-vaccination Ab responses have extended to human studies. Indeed, higher magnitude female B-cell responses have been documented in a cohort of 138 adults aged 50–74, as were higher numbers of CD4+ T-cells and fewer NK cells [281]. Another recent study reported that human females mounted higher Ab and IL-6 responses after H1N1 vaccination [282]. A study of vaccinated healthcare workers within Johns Hopkins Hospital (*n* = 274) did not observe overall significant differences in neutralizing Ab titers between males and females when all age groups were merged [283]. Females in older age groups had higher titers than males. Elevated female body mass index resulted in reduced Ab responses; males did not exhibit the same trend [283].

Sex hormones influence immune responses to influenza vaccination. Human nasal epithelial cell cultures were treated with endogenous 17-estradiol and estrogen receptor modulators to analyze their influence on influenza A viral replication [284]. Cell lines derived from female donors had reduced IAV titers when they were exposed to bisphenol A, estradiol, or raloxifene. Genomic estrogen receptor-2 was required for the viral titer to decrease. Cell lines derived from male donors did not have the same effect, indicating researchers also need to consider testing cell lines derived from both sexes in preclinical studies. Higher concentrations of the sex hormone estradiol correlated with higher Ab responses to influenza vaccination in female humans [282]. Moreover, C57BL/6 female mice had more protective responses to H1N1 vaccinations, although the effect diminished in older mice. Administering estradiol increased Ab responses, whereas administering testosterone diminished the responses [282]. Thus, estradiol treatment post-influenza vaccination may boost memory responses and protect a larger proportion of susceptible population subsets.

Testosterone was characterized as having an immunosuppressive influence after influenza vaccination [285]. In this study with 87 participants, female serum had higher concentrations of inflammatory cytokines and Abs, which correlated with phosphorylated STAT3 in monocytes [285]. This provides a mechanistic explanation for why females tend to have lower viral titers during influenza infections but a propensity towards more severe inflammation-mediated pathogenesis. Males with higher levels of testosterone had lower titers of antibodies induced by the trivalent inactivated influenza vaccine. Testosterone has been shown to have an immunosuppressive effect by interacting with the androgen receptor on CD4+ T-cells to produce the immunosuppressive cytokine IL-10 [286]. Male SJL mice also produce more IL-4 and reduced quantities of IL-12 compared to female mice [286]. IL-4 promotes naïve T-cells to differentiate into the Th2 subtype, which is less favorable than the Th1 response for effective viral clearance. Testosterone levels decrease with age, which may explain higher incidences of immune-mediated severe influenza in senior males due to excessive inflammation in response to the infection [287]. Indeed, higher testosterone levels proved to be protective against inflammatory damage following H1N1 influenza infection in a C57BL/6 mouse model [288]. Similarly, treating female mice with testosterone reduced influenza-mediated mortalities by preventing inflammatory responses from becoming excessive.

A mounting body of evidence exists that sexual dimorphism might contribute substantially to influenza vaccination responses and, thus, should always be considered when designing the next generation of influenza vaccines. Males generally produce fewer neutralizing Abs in response to modern vaccines and have more severe infections in elderly populations. Thus, consideration should be given to boosting male immunity in senior populations. Developing sex-optimized vaccines would contribute to lowering the mortality of annual influenza infection in hospitals and long-term care facilities.

## 14. Conclusions

Influenza viruses remain a global problem, with no permanent solution. Annual vaccines are currently used to manage influenza infections. Yet seasonal influenza results in hundreds of thousands of deaths annually, with varying vaccine efficacy each year [289]. The development of new methods to design vaccines that cater to the more-qualitative aspects of immunological responses could assist in the production of more effective vaccines, and possibly a more-universal type of vaccine for influenza. The qualitative aspects of the host immune systems are of even more crucial consideration in immunocompromised populations, such as young children, elderly people, and individuals with underlying health conditions, where influenza is a more serious health concern [240]. The qualitative aspects of host immune responses, including trained immunity, should be exploited in vaccine design to optimize immunity against influenza, and confer a broader, longer lasting, and more diverse range of responses.

## Data Availability

Not applicable.
